# Effects of Shared Electronic Health Record Systems on Drug-Drug Interaction and Duplication Warning Detection

**DOI:** 10.1155/2015/380497

**Published:** 2015-11-22

**Authors:** Christoph Rinner, Wilfried Grossmann, Simone Katja Sauter, Michael Wolzt, Walter Gall

**Affiliations:** ^1^Center for Medical Statistics, Informatics and Intelligent Systems, Medical University of Vienna, 1090 Vienna, Austria; ^2^Research Group Scientific Computing, University of Vienna, 1090 Vienna, Austria; ^3^Department of Clinical Pharmacology, Medical University of Vienna, 1090 Vienna, Austria

## Abstract

Shared electronic health records (EHRs) systems can offer a complete medication overview of the prescriptions of different health care providers. We use health claims data of more than 1 million Austrians in 2006 and 2007 with 27 million prescriptions to estimate the effect of shared EHR systems on drug-drug interaction (DDI) and duplication warnings detection and prevention. The Austria Codex and the ATC/DDD information were used as a knowledge base to detect possible DDIs. DDIs are categorized as severe, moderate, and minor interactions. In comparison to the current situation where only DDIs between drugs issued by a single health care provider can be checked, the number of warnings increases significantly if all drugs of a patient are checked: severe DDI warnings would be detected for 20% more persons, and the number of severe DDI warnings and duplication warnings would increase by 17%. We show that not only do shared EHR systems help to detect more patients with warnings but DDIs are also detected more frequently. Patient safety can be increased using shared EHR systems.

## 1. Introduction

One of the main advantages of a shared electronic health record (EHR) system [1], a system that shares patients clinical information in an electronic form across institutions, is to quickly offer access to all available clinical information on a patient at the point of care. It also has other benefits such as improvement in quality of care, patient safety, increased efficiencies, and cost savings [2]. For patients receiving shared treatment, the availability of a complete medication overview of a patient's prescriptions is a key feature for decision support and medication safety [3].

According to the OECD study on the role of information and communication technologies, easily accessible information for reconciling the medication prescribed is one fundamental for improving medication quality [4]. Reconciliation of a patient's medications using electronic medication records can support finding consensus between healthcare providers (HCP) and help them to detect possible adverse drug events (ADEs). Providers of primary care and hospitals especially can benefit [5].

However, reviews still show a lack between the promised and the empirically demonstrated effects that e-Health services offer for the safety of health care delivery [6]. Even if e-Health services like the e-Prescription, a service for physicians to electronically send prescriptions to pharmacies, are widely used in health care, there is a further need to provide evidence of positive effects like cost-effectiveness [7].

Austria is introducing a nationwide shared EHR system called ELGA in 2015 [8] which is based on the Integrating the Healthcare Enterprise (IHE) Cross-Enterprise Document Sharing (XDS) profile [9]. Besides laboratory reports, radiology reports, and hospital discharge letters, a patient medication history including all prescriptions of a patient is planned. This service as part of ELGA, called “e-Medikation,” will start in 2016. In the context of this study, e-Medikation refers to the planned ELGA-service that gives an electronic overview of a patient's medication history including the prescriber and dispenser of a prescription. It enables physicians as well as pharmacists to enter new prescriptions and over-the-counter drugs and is a variation of e-Prescription. A prescription refers to the number of packages of a single drug prescribed to a specific patient, at a specific time, and from a specific HCP. Although in Austria, like in other countries, a positive attitude towards e-Health and e-Prescription exists in general [10], the planned e-Medikation system is controversially debated on the healthcare provider side (i.e., accountability, information overload, and usability) and on the patient side (i.e., security and data privacy concerns) [11].

There is a trend towards e-Prescription all over Europe. In some countries, systems are already established as in Scandinavia, England, or the Netherlands. For example, Denmark and Sweden already started in 2002 with the introduction of a nationwide electronic prescription system [12]. In other countries, systems are planned as in Germany or as mentioned in Austria. The systems currently in use and planned have different goals and architectures: mostly e-Prescription systems are used as in Sweden [13], the Netherlands [14], or England [15]. In Turkey, a web-based solution should support pharmacists [16]. Germany is going to introduce a standardized and paper based medication plan (German “Medikationsplan”) that covers the patient's entire medication [17].

Drug-drug interactions (DDIs) are an important cause of ADEs [18]. The number of physicians involved in the management of a patient is an important risk factor [19] leading to inappropriate and duplicate medication. “Doctor shopping,” the change of physicians without referral, which is possible and common in many health systems [20], acts as an intensifier. Clinical decision support systems for medication safety are considered helpful but can increase the workload and disrupt the workflow of health care professionals due to inflation of warnings [21]. Particularly, warnings of duplicate drug prescriptions often result in overrides [22]. To reduce alert fatigue DDIs should be noninterruptive and often a small number of interactions account for a large number of interruptions [23]. Detailed analyses on the kind and the amount of warnings are necessary to develop intelligent systems to support different professionals in the treatment of different patient groups in different settings.

Access to (reimbursed) medication data in the form of health claims data offers an alternative to future medication data of shared EHR systems. Many countries use reimbursement data sources for pharmaceutical clinical studies; for example, Nordic countries have a long tradition [24]. In Denmark, drug prescriptions have been recorded nationwide in a central register since 1994 [25]. These databases can be used for the identification of new effects of drugs and the appropriateness in drug use [26]. For our study we use a similar database with health claims data to estimate the number of interaction and duplication warnings that patients theoretically release. The “General Approach for Patient-oriented Diagnoses Related Groups” (GAP-DRG) database is maintained by the Main Association of Austrian Social Security Institutions and made available for research purposes. Besides dispensed medications, it contains hospital stays and related diagnoses as well as sociodemographic attributes from 2006 to 2007.

The upcoming Austrian nationwide e-Medikation system allows physicians to retrieve information about the entire medication of their patients (covering drug prescriptions they and other HCPs made), which as a consequence should prevent DDIs and reduce ADEs. In a previous study we developed a tool to explore possible ADEs using health claims data [27] and analysed the effect of severe DDIs in the context of additional workload on healthcare professionals depending on the health care professional group [28].

Shared EHR systems appear promising, but the advantages need to be justified. Studies report that they are perceived to improve the safety in health care because they provide a more complete medical history [29]. Nowadays, several European countries have implemented an e-Prescription system as part of a shared EHR system [12], while in Austria the system called “e-Medikation” is planned by 2016. Currently the subject of controversial debates, a statement about the cost-benefit analysis of the e-Prescription is difficult. Studies of e-Health and e-Prescription systems show a very uneven distribution of cost and benefits among the participations [30, 31]. Besides the expected benefits, improper use of EHR systems and ignoring information integrity in shared EHRs can also lead to new types of patient safety hazards and hence decrease patient safety [32]. e-Medikation using shared EHR systems is intended to improve patient hand-over effectiveness [33] and thus increase patient safety.

This study focuses on the patient, with the goal of estimating the effect of the introduction of the Austrian nationwide e-Medikation system on the number of DDI warnings and duplication warnings depending on the age group. A method was developed that integrates medication specific information about possible interactions with other drugs and intake habits according to the ATC.

## 2. Methods

### 2.1. Study Population

The amount of expected DDI warnings and duplication warnings is estimated using the research database of the Main Association of Austrian Social Security Institutions. The research database contains pseudonymized health claims data of all Austrian public health insurance companies of the years 2006 and 2007, covering about 7.9 million patients and 95% of the Austrian population. Since the temporal proximity of the prescribed drugs is essential for the estimation of DDI warnings and duplication warnings, only patients which are insured by the three health insurance companies that document the exact date of the dispensation were selected; hence our study cohort is reduced to about 1.3 million patients. The study cohort was further restricted to patients between the ages of 20 and 99 and having at least one prescription in the one-year time period between July 1, 2006, and June 30, 2007, that we focused on, resulting in 1.043.762 patients.

As shown in Table 1, the study cohort covers about 16% of the Austrian population of 2007 (according to Austrian Office for Statistics “Statistik Austria” [34]). In three rural provinces (Lower Austria, Carinthia, and Salzburg), more than 40% of the population is covered. In Vienna, the most urban region in Austria, 6.7% of the population is contained in the study cohort; in the other 5 provinces, less than 1.1% of the population is covered. In our study cohort, 40.6% are male and 58.4% are female, while for 1% the gender is unknown (neither male nor female).

**Figure 1:**
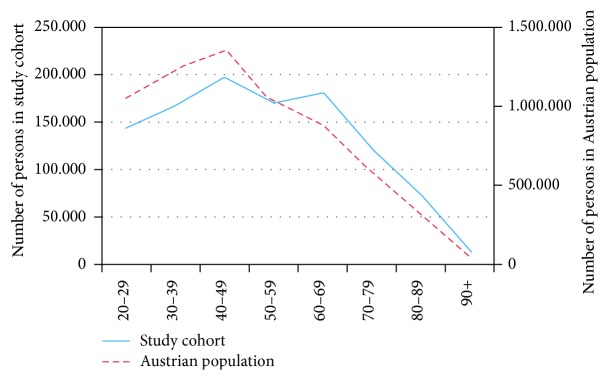
Age distribution of the study cohort compared to the population of Austria on the reporting date of January 1, 2007.

In Figure 1, the age distribution of our study cohort compared to the age distribution in Austria is shown. In both cohorts, the number of persons is the highest in the age group of 40 to 49 years. In our cohort we have an additional peak at 60 to 69 years. Our study cohort is slightly older than the Austrian population. In Austria the population in the western provinces and the population in the cities are slightly younger. Since our cohort mainly covers the eastern provinces, this shift could be explained.

### 2.2. Prescriptions

Our study cohort of about 1 million persons received a total of about 27 million prescriptions in the time period between February 15, 2006, and June 30, 2007 (one year and 4.5 months of lead time), that we used to calculate our DDI and duplication warnings. In Table 2 the number of persons and the number of prescriptions in general, as well as the prescriptions per person per year grouped by age group, are listed. As seen in Table 2, the older the patients in our study cohort are, the more the number of prescribed prescriptions increases. Starting with a median of 4 prescriptions a year in the age group of 20 to 29 years, a person of the age group of 90+ received in median 59 prescriptions.

### 2.3. Calculation of Warnings

In order for two prescriptions to result in a DDI warning or duplication warning, one or both prescriptions must have a dispensing date in the observed one-year time period of July 1, 2006, and June 30, 2007. To ensure that we find simultaneous intake of two drugs also at the beginning of our one-year observation time, we added 4.5 months (i.e., threshold for maximum theoretical duration of intake) lead time before the observation period. In this lead time, the first of the two prescriptions could have been prescribed, yet the actual ADE occurs in the one-year observation time, when the second prescription is prescribed.

Potential safety warnings were assessed on the basis of interactions of the active pharmaceutical ingredients (API). These API are categorized in the 5th level of the international ATC code. The unique Austrian pharmaceutical registration number (PRN) in the GAP-DRG database was used for identification of package size and thus potential overlap of prescribed medications. The 26,922,874 prescriptions are assigned to 11,668 different pharmaceutical products identified by a unique PRN. 11% of the prescriptions could not be used to calculate DDI and duplications warnings, because their PRN was not available in the database. They refer, for example, to individual magistral preparation by pharmacies, blood glucose test strips, infusion accessories, or tube feeding supplements.

For each prescription, the theoretical duration of intake was calculated using the ATC-DDD classification [35]. Under consideration of the number of prescribed packages and the defined daily dose (DDD), a specific period of time is calculated that needs to overlap with the prescription of the second drug of the same patient. Medicinal products exist in various package sizes, which can be deduced from the pharmaceutical number. The pharmaceutical number is essential; it is listed in the Austria Codex and is used for reimbursement and therefore is available in the GAP-DRG database. The DDD is a statistical number, based on recommendations of pharmaceutical manufacturers. It is not possible to calculate it for epidermal surfaces and it is further not available for rare diseases. In case it was not possible to calculate a theoretical duration of intake (i.e., no DDD was assigned to the ATC code), a period of 30 days was assumed and assigned to the prescription. If the time periods of two prescriptions overlapped, DDI and duplication warnings are checked.

Depending on the drug, a prescription has exactly one ATC code assigned. Using the Austria Codex [36], the ATC codes are allocated to substance groups which are used to calculate a DDI warning. The Austria Codex categorizes DDI warnings into severe, moderate, and minor interactions. Severe DDIs may cause permanent damage or may be life-threatening, while moderate DDIs result in potential deterioration of the patient's condition whereas minor DDIs only result in mild adverse effects.

If a drug-drug interaction results in more warnings of different severity levels, only the warning with the highest level is counted. A duplication warning is assumed if the same drug (i.e., same ATC code) is prescribed more than once within a time period. For the calculation of the duplication warnings, half of the theoretical duration was used as tolerance time because patients might receive supply of a medication.

### 2.4. With and without e-Medikation

The Austrian e-Medikation system will enable HCPs to see the entire medication list of a patient, which also includes the prescriptions from colleagues. To estimate the effect of the e-Medikation on the number of DDI and duplication warnings, the prescriptions from all HCPs are used.

If the drug-drug combination resulting in a DDI or duplication warning was prescribed by the same HCP, the current system without e-Medikation is assumed. For the calculation of the warnings in a future system with e-Medikation, the prescriber of the first drug and the prescriber of the second drug can differ. Using this method it is possible to distinguish between a system where information from only a single HCP is available compared to a system where information from different HCPs is available.

In a shared EHR system with e-Medikation, DDI warnings are calculated using the entire medication of a patient of different HCP, while in a system without e-Medikation only prescriptions that were issued by one health care provider is considered.

### 2.5. Statistical Methods

Data were cleaned and preprocessed using the PostgreSQL database version 9.1.3. Statistical analyses were performed with R and visualized with SPSS.

## 3. Results

### 3.1. Prescriptions with Warnings

First we analysed the prescriptions affected by DDI and duplication warnings with and without e-Medikation. Since prescriptions from other HCPs can be used to calculate the DDI and duplication warnings, the number of prescriptions with warnings increases. Tables 3 and 4 show the number of prescriptions with severe and moderate DDI warnings, respectively, and prescriptions with minor DDI and duplication warnings with and without e-Medikation per age group.

It has to be mentioned that the younger age groups have fewer warnings (Table 2), but a higher increase of prescriptions is estimated (Tables 3 and 4). For example, the increase of prescriptions with severe DDI warnings for all persons is 17.4%; for people in the age group 30–39 it is 26.6%.

### 3.2. Persons with Warnings

Tables 5 and 6 show the number of persons with severe and moderate DDI warnings, respectively, the minor DDI warnings, and the duplication warnings with and without e-Medikation per age group. The number of persons with DDI and duplications warnings increases with e-Medikation for all severity levels and age groups. The number of persons with severe DDI warnings increases from 11,217 to 13,483 (increase of 20%) over all age groups. The highest increase for severe DDI warnings is measured in the age group of 30–39 years with 30.7%. The same effect of the e-Medikation can be seen with moderate and minor DDI warnings as well as duplication warnings.

**Figure 2:**
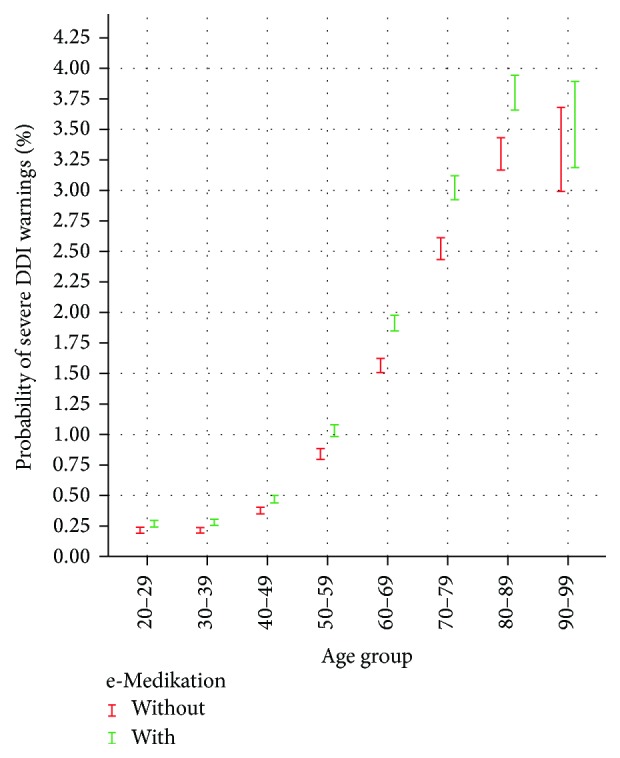
Confidence interval of probability of a severe DDI warning without (drugs issued by one HCP) and with e-Medikation (drug issued by different HCPs) per age group.

For the analysis of the differences in the proportions, we computed confidence intervals for persons with and without e-Medikation. We used the procedure “binom.test” in R for the computation of the 0.95 confidence intervals, which gives exact confidence intervals using the method of Clopper and Pearson [37]. This method guarantees that the intervals have at least the confidence level and avoid problems in case of normal approximation for small proportions. Because our approach is more oriented towards descriptive statistics, we prefer confidence intervals instead of tests for the different age groups.  Figure 2 shows the results in the different age groups for severe DDI warnings. As one can see, there is a nonlinear increase in the proportions of warnings over the age groups up to 89 years. The nonoverlapping confidence intervals from the age groups of 40–49 up to 80–89 years show that there is a significant increase in the proportions with e-Medikation compared to the groups without e-Medikation at the 5% significance level.

Besides the proportions of persons affected by DDI warnings, it is also of interest to analyse the number of warnings per person with and without e-Medikation. As a model for the number of DDI warnings, we used a Poisson distribution and assumed that the parameter of the Poisson distribution is the same for each person within an age group. This parameter can be interpreted as the intensity for the number of warnings per year within each age group, that is, the average number of warnings per year for the age group. Under this assumption, the estimate of the intensity is simply the average overall person counts and confidence intervals for the estimates can be computed. Similar to the proportions of warnings, 0.05-confidence intervals were computed for the estimates in the different age groups for persons with and without e-Medikation.

**Figure 3:**
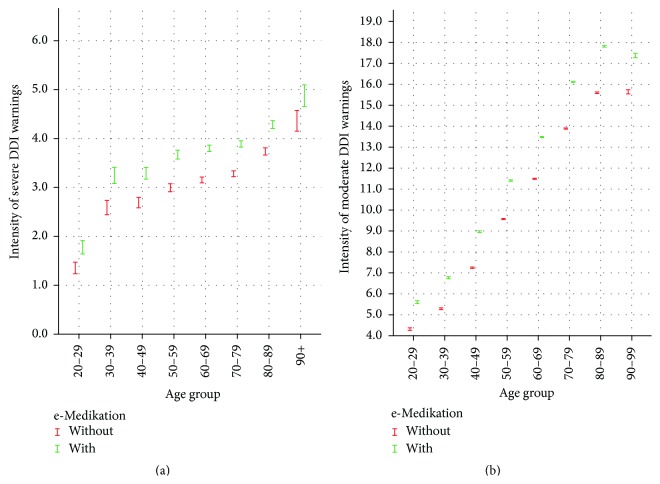
Confidence interval of intensity of severe and moderate DDI warnings without (drugs issued by one HCP) and with e-Medikation (drug issued by different HCPs) per age group.

**Figure 4:**
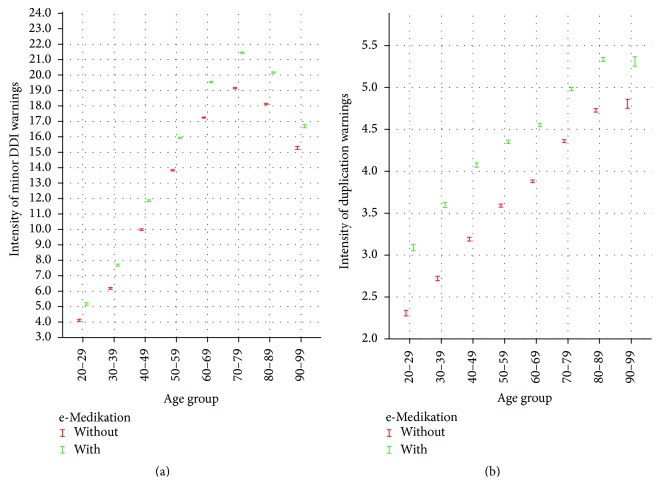
Confidence interval of intensity of minor DDI and duplication warnings without (drugs issued by one HCP) and with e-Medikation (drug issued by different HCPs) per age group.

Figures 3 and 4 show the confidence level with and without e-Medikation in the different age groups for severe and moderate DDI warnings, respectively, and minor DDI and duplication warnings. The graphics show clearly that the intensity of warnings per person increases significantly at the 5% significance level due to e-Medikation in all age groups. In other words, not only does e-Medikation detect more patients with warnings, but also the detection is more frequent per patient.

## 4. Discussion 

Our analysis shows clear indications that the Austrian e-Medikation has the potential to increase patient safety by increasing the number of warnings and reducing expenses for duplicate drug prescriptions. In all age groups, an increase of DDI warnings with e-Medikation compared to no e-Medikation could be measured, yet the effect varies significantly between the different age groups. The highest increase for severe DDI warnings is observed in the age group of 30–39 years with over 30% more patients affected.

Comparisons of our results with other countries are difficult due to the fact that the health care systems are different and also there are a variety of study designs. In our study design, if a drug-drug combination results in more warnings with different severity levels, only the highest level is counted. Hence the numbers of minor interaction warnings, which could occur simultaneously, are underestimated compared to severe or moderate ones.

We used health claims data as our data source. Such data sources have great potential for the usage in studies about patient safety. On the other hand, they have several limitations. In our case, the limitations were the lack of information about medication given during hospital stays and also over-the-counter medicine that is not reimbursed by health insurance companies. Also, our study design reduced our study collective to about 1 million persons that mostly lived in rural areas.

The database information about DDIs in the Austria Codex is compiled by ABDATA Pharma-Daten-Service in Germany and includes information from the summary of product characteristics and expert-adjudicated literature research. The severity of a DDI is graded accordingly, yet the clinical relevance of DDIs may be limited. We have recently reported that ADEs may be present in approximately 1.5% of hospitalised patients of the GAP-DRG database [38]. Of these, a DDI was identified in 68% (13,511 subjects) and a severe interaction in 12% (2,412 subjects), respectively. In the absence of a control group, the descriptive nature of a DDI does not allow for risk quantification or qualification of the clinical relevance of drug interactions. Ongoing documentation of DDI occurrence will have to be combined with clinical data to refine the classification of severity of interactions and to limit the number of warnings accordingly.

Health claims data are gaining importance in pharmacoepidemiological studies. Often the ATC/DDD information is used to calculate drug behaviours of the patients and potential DDIs [39]. We are using health claims data to analyse the prescriptions affected by DDI and duplications warnings with and without e-Medikation per age group to quantify the expected benefits of spending money to share medication data in shared EHRs. We categorized DDI warnings into severe, moderate, and minor and deduce the number of affected persons and do not focus on improvement of efficiencies or drug cost savings as shown in [31].

Besides the general critical success factors for the introduction of an e-Medikation system mentioned in [40], the refinement of knowledge can increase the quality of the warnings. Our study offers insights into the expected numbers of DDI warnings and affected persons in a population not focused on in isolated areas when consolidating prescribing information. This allows quantification of hand-over effectiveness which is mentioned as an indicator for patient safety [33].

A future DDI warning system has to take into account the different age groups, medical specialist groups, and groups of medications prescribed and individually adapted to specific needs.

Checking DDIs with all drugs of a patient compared to only the drugs prescribed by a single health care provider, the number of patients affected as well as the number of DDI and duplication warnings increases in all age groups.

## 5. Conclusions

In this study we used health claims data from the Austrian Social Security Institutions to deduce the effect of shared EHR systems on the number of DDIs and duplications warnings. Our method of using health claims data can also be applied in epidemiological research. As a next step, we plan to analyse the detected DDI and duplication warnings with a medical focus to additionally categorize medication combinations or application spectrums particularly affected by the introduction of an e-Medikation system. This needs a combination of the e-Medikation data with data about ADE, which will allow the computation of sensitivity and specificity of the DDI detection instrument. Another topic of further research is the development of models for the increase of DDI interaction with special focus on different age groups.

## Figures and Tables

**Table 1:** The study cohort compared to the population of Austria on the reporting date of January 1, 2007.

Province	Population 1.1.2007^*∗*^	Population older than 20 years^*∗*^	Persons in cohort older than 20 years and exact prescription date	% of persons in cohort compared to population
Lower Austria	1,592,500	1,244,330	518,232	41.65%
Carinthia	559,829	441,755	207,095	46.88%
Salzburg	525,826	407,494	198,689	48.76%
Vienna	1,665,458	1,338,391	89,283	6.67%
Styria	1,203,132	956,725	9,629	1.01%
Upper Austria	1,405,127	1,082,401	7,954	0.73%
Tyrol	697,863	538,733	7,798	1.45%
Burgenland	280,469	225,451	1,863	0.83%
Vorarlberg	364,985	275,846	1,055	0.38%
No province			2,164	

Sum	8,295,189	6,511,126	1,043,762	16,03

^*∗*^According to Statistik Austria.

**Table 2:** Number of persons and prescriptions per person in study cohort.

Age group	Persons in study cohort	Prescriptions in study cohort	Prescriptions per person and year			
			1st quarter	Median	Mean	3rd quarter
20–29	140,961	893,383	2	4	6.3	7
30–39	164,533	1,439,362	2	4	8.8	9
40–49	194,053	2,530,847	3	6	13.0	14
50–59	167,264	3,961,860	5	13	23.7	29
60–69	177,894	6,459,356	10	24	36.3	49
70–79	118,528	6,209,350	19	40	52.4	73
80–89	69,785	4,651,582	29	56	66.7	92
90+	10,744	777,134	31	59	72.3	99

Sum	1,043,762	26,922,874				

**Table 3:** Prescriptions with severe and moderate DDI warnings without (drugs issued by one HCP) and with e-Medikation (drug issued by different HCPs) per age group.

Age group	Number of prescriptions	Severe DDI warnings						Moderate DDI warnings					
		Without e-Medikation		With e-Medikation		Increase		Without e-Medikation		With e-Medikation		Increase	
		*n*	%	*n*	%	*n*	%	*n*	%	*n*	%	*n*	%
20–29	893,383	869	0.10	1,093	0.12	224	25.8	21,325	2.39	26,330	2.95	5,005	23.5
30–39	1,439,362	1,423	0.10	1,801	0.13	378	26.6	63,153	4.39	76,363	5.31	13,210	20.9
40–49	2,530,847	3,176	0.13	3,853	0.15	677	21.3	186,814	7.38	218,108	8.62	31,294	16.8
50–59	3,961,860	6,639	0.17	7,916	0.20	1,277	19.2	410,905	10.37	468,481	11.82	57,576	14.0
60–69	6,459,356	13,898	0.22	16,546	0.26	2,648	19.1	739,927	11.46	831,723	12.88	91,796	12.4
70–79	6,209,350	15,513	0.25	18,239	0.29	2,726	17.6	796,121	12.82	886,839	14.28	90,718	11.4
80–89	4,651,582	13,020	0.28	14,773	0.32	1,753	13.5	588,190	12.64	646,178	13.89	57,988	9.9
90–99	777,134	2,170	0.28	2,353	0.30	183	8.4	83,437	10.74	89,631	11.53	6,194	7.4

Sum, mean	26,922,874	56,708	0.21	66,574	0.25	9,866	17.4	2,301,682	8.55	3,217,323	11.95	282,583	12.3

**Table 4:** Prescriptions with minor DDI and duplication warnings without (drugs issued by one HCP) and with e-Medikation (drug issued by different HCPs) per age group.

Age group	Number of prescriptions	Minor DDI warnings						Duplication warnings					
		Without e-Medikation		With e-Medikation		Increase		Without e-Medikation		With e-Medikation		Increase	
		*n*	%	*n*	%	*n*	%	*n*	%	*n*	%	*n*	%
20–29	893,383	14,963	1.67	18,314	2.05	3,351	22.4	30,062	3.36	39,569	4.43	9,507	31.6
30–39	1,439,362	42,448	2.95	50,788	3.53	8,340	19.7	63,888	4.44	82,875	5.76	18,987	29.7
40–49	2,530,847	143,042	5.65	163,997	6.48	20,955	14.7	133,442	5.27	167,190	6.61	33,748	25.3
50–59	3,961,860	398,569	10.06	446,535	11.27	47,966	12.0	236,724	5.98	284,046	7.17	47,322	20.0
60–69	6,459,356	827,639	12.81	910,074	14.09	82,435	10.0	406,793	6.30	473,916	7.34	67,123	16.5
70–79	6,209,350	900,417	14.50	981,409	15.81	80,992	9.0	412,262	6.64	469,396	7.56	57,134	13.9
80–89	4,651,582	643,191	13.83	696,134	14.97	52,943	8.2	323,523	6.96	363,580	7.82	40,057	12.4
90–99	777,134	88,489	11.39	94,279	12.13	5,790	6.5	53,181	6.84	58,589	7.54	5,408	10.2

Sum, mean	26,922,874	3,058,758	11.36	3,361,530	12.49	330,250	10.8	1,659,875	6.17	1,408,391	6,58	279,286	16.8

**Table 5:** Persons per age group with severe and moderate DDI warnings without (drugs issued by one HCP) and with e-Medikation (drug issued by different HCPs).

Age group	Persons in cohort	Severe DDI warnings						Moderate DDI warnings					
		Without e-Medikation		With e-Medikation		Increase		Without e-Medikation		With e-Medikation		Increase	
		*n*	%	*n*	%	*n*	%	*n*	%	*n*	%	*n*	%
20–29	140,961	302	0.2	377	0.3	75	24.8	3,511	2.5	4,491	3.2	980	27.9
30–39	164,533	352	0.2	460	0.3	108	30.7	9,229	5.6	11,174	6.8	1,945	21.1
40–49	194,053	728	0.4	910	0.5	182	25.0	22,254	11.5	25,743	13.3	3,489	15.7
50–59	167,264	1.404	0.8	1,724	1.0	320	22.8	40,130	24.0	44,781	26.8	4,651	11.6
60–69	177,894	2.784	1.6	3,401	1.9	617	22.2	61,751	34.7	67,538	38.0	5,787	9.4
70–79	118,528	2.989	2.5	3,581	3.0	592	19.8	56,816	47.9	60,914	51.4	4,098	7.2
80–89	69,785	2.301	3.3	2,651	3.8	350	15.2	38,864	2.5	40,983	58.7	2,119	5.5
90–99	10,744	357	3.3	379	3.5	22	6.2	5,679	5.6	5,901	54.9	222	3.9

Sum, mean	1,043,762	11,217	1.1	13,483	1.3	2,266	20.2	198,104	22.8	261,525	25,1	23.291	11,8

**Table 6:** Persons per age group with minor DDI and duplication warnings without (drugs issued by one HCP) and with e-Medikation (drug issued by different HCPs).

Age group	Persons in cohort	Minor DDI warnings						Duplication warnings					
		Without e-Medikation		With e-Medikation		Increase		Without e-Medikation		With e-Medikation		Increase	
		*n*	%	*n*	%	*n*	%	*n*	%	*n*	%	*n*	%
20–29	140,961	2,819	2.0	3,541	2.5	722	25.6	6,238	4.4	8,002	5.7	1,764	28.3
30–39	164,533	5,627	3.4	7,004	4.3	1,377	24.5	11,652	7.1	14,430	8.8	2,778	23.9
40–49	194,053	13,501	7.0	15,947	8.2	2,446	18.1	21,724	11.2	25,585	13.2	3,861	17.8
50–59	167,264	29,794	17.8	34,222	20.5	4,428	14.9	35,211	21.1	39,919	23.9	4,708	13.4
60–69	177,894	52,371	29.4	57,937	32.6	5,566	10.6	57,338	32.2	63,183	35.5	5,845	10.2
70–79	118,528	51,561	43.5	55,186	46.6	3,625	7.0	52,684	44.4	56,778	47.9	4,094	7.8
80–89	69,785	37,495	53.7	39,240	56.2	1,745	4.7	38,291	54.9	40,649	58.2	2,358	6.2
90–99	10,744	5,765	53.7	5,962	55.5	197	3.4	6,274	58.4	6,547	60.9	273	4.4

Sum, mean	1,043,762	198,933	19.1	219.039	21.0	20,106	10.1	229,412	22.0	255.093	24.0	25,681	11.2
